# Predicting orbital fractures in head injury: a preliminary study of clinical findings

**DOI:** 10.1007/s10140-019-01720-0

**Published:** 2019-08-30

**Authors:** James R. Allison, Andrew Kearns, Robert J. Banks

**Affiliations:** 1 Clinical Fellow in Oral Surgery, School of Dental Sciences, Newcastle University, Framlington Place, Newcastle upon Tyne, NE2 4BW UK; 2 Manchester University, Manchester, UK; 3 Sunderland Royal Hospital, Sunderland, UK

**Keywords:** Head trauma, Fracture, orbital, Signs and symptoms, X-ray computed tomography, Maxillofacial injuries

## Abstract

**Purpose:**

Patients presenting to emergency departments (EDs) following head injury often undergo computed tomography (CT) of the head to exclude traumatic brain injury. In many cases, this does not show the maxillofacial skeleton. A proportion of these patients also sustain facial fractures, and when fractures involve the orbits, CT imaging is useful in diagnosis and management; obtaining a second scan may cause delay, incur greater cost, and increase radiation dose. The aim of this preliminary study was to examine the value of signs and symptoms of orbital fractures in predicting a fracture on CT.

**Methods:**

The clinical records of 47 patients who underwent CT of the face following facial trauma were retrospectively examined for the presence of signs and symptoms of orbital fractures. Sensitivity, specificity, negative predictive value (NPV) and positive predictive values (PPV) were then calculated for each sign and symptom for the presence of an orbital fracture on CT. We also described a clinical decision instrument and examined the predictive values of this.

**Results:**

Change in the position of the globe, reduced visual acuity, subconjunctival haemorrhage and change in sensation in the maxillary division of the trigeminal nerve were the most specific signs and symptoms for orbital fracture. Our clinical decision instrument had 80.0% sensitivity, 75.0% specificity, 90.3% PPV and 56.3% NPV for predicting the presence of an orbital fracture on CT in this population.

**Conclusions:**

Our results demonstrate that signs and symptoms of orbital fractures may be useful for predicting these injuries, and a decision instrument could be used in the ED to identify patients likely to benefit from extending the radiation field to include the orbits where CT of the head is already planned. This work is however exploratory; and further prospective validation is required before a robust instrument can be recommended for clinical use.

## Introduction

**Fig. 1:**
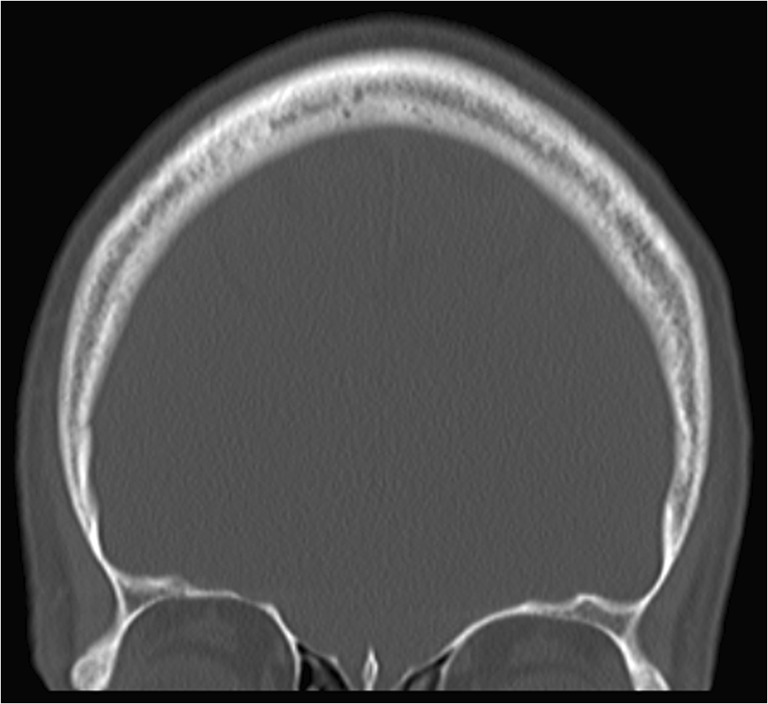
Field of view often seen in computed tomography imaging taken to exclude brain injury following head trauma

There were over 21 million attendances to emergency departments (EDs) in hospitals in England between April 2017 and March 2018, and in over 443,758 cases the first recorded diagnosis was “head injury” [1]. Many of these patients will also have had fractures of the facial skeleton, as these injuries are seen in a significant proportion of patients who present with head injury [2]. Decision-making rules have been well described for performing radiographic investigations in suspected fractures of the cervical spine [3], knee [4] and ankle [5], and these are widely used clinically. Although well-recognised guidance exists to identify patients who are likely to benefit from computed tomography (CT) imaging of the head and neck to exclude injury to the brain and cervical spine [6], this often does not include the facial skeleton [7] (Fig. 1). Although true for isolated head injury, this is likely to be a less significant problem in major trauma, where whole body CT is more common [8].

CT is an indispensable modality for the diagnosis and management of facial trauma [9], and this is particularly true of fractures of the orbit due to the complex local anatomy, which is not usually well demonstrated using plain film techniques [10]; additionally, CT is useful in identifying complications of orbital fractures such as entrapment of the extraocular muscles and orbital compartment syndrome resulting from retrobulbar haemorrhage or haematoma [11, 12]. Orbital fractures are present in 5.3–19.7% of patients presenting with head injury depending on the population studied [7, 13]. It is often the case that when patients with suspected orbital trauma are examined by members of specialty teams, patients have already undergone CT of the head. If a fracture of the orbit is subsequently suspected on the basis of examination, these patients often go on to have additional imaging which causes delay in diagnosis and discharge from the ED, as well as increased cost and service use compared with if the images were obtained at the same time as head CT. Obtaining two scans also incurs greater radiation dose: the effective doses for common CT investigations in the authors’ unit can be seen in Table 1 and show that where imaging of the orbits is included with CT of the head, this incurs a 0.48 milliSieverts (mSv) lower dose than if separate scans of the head and orbits are taken. Where the field is further extended to include all of the facial bones at the same time as head CT, the dose is 0.48–0.63 mSv lower than if head CT and CT of the facial bones are taken separately. As a comparison, the average annual background radiation dose in the UK is 2.7 mSv [14].

**Table 1:** Effective doses for common CT investigations. Calculated using reference dose length product (DLP) for equipment used in the Radiology Department of Sunderland Royal Hospital as per Shrimpton et al. [26]

Investigation	Effective dose (mSv)
CT head	1.26
CT orbits	1.32
CT facial bones	1.26–1.47
CT head and facial bones	< 2.1

The aim of the present, exploratory study was to examine the value of signs and symptoms of orbital trauma for predicting the presence of an orbital fracture on CT. The objective was to identify those signs and symptoms with high predictive value that could then be used in future prospective studies, to allow a robust decision instrument to be devised which could be used clinically to identify patients likely to benefit from including the orbits if a CT of the head was already planned. This would mean that appropriate imaging would be available to specialist teams when called to review the patient, and allow expedient diagnosis, treatment decisions, and discharge from the ED to be performed at reduced radiation dose to the patient and demand on services.

## Methods

The picture archiving and communication system of Sunderland Royal Hospital, a district general hospital in the north east of England, was retrospectively searched between 1st of January 2016 and 7th of June 2017, to identify patients who received any CT investigation which included the orbits following presentation with trauma to the face. This 18-month period was chosen as a convenience sample.

A total of 67 patients received a CT investigation which included the orbits following facial trauma between 1st of January 2016 and 7th of June 2017. Twenty were excluded because either the notes were of insufficient quality to assess the presence of the signs and symptoms of interest, or the initial presentation following trauma was not within the hospital, and the notes from first presentation were therefore unavailable. Forty-seven patients were therefore included.

Patients’ electronic medical records were reviewed by two investigators (JA, AK), and the clinical notes from the initial assessment at the patients’ first presentation were used to record the presence or absence of the signs and symptoms described in Table 2*.* Where the notes from the initial assessment did not include sufficient information, or the initial presentation was not within the study site and notes were therefore unavailable, these patients were excluded. Radiological reports and CT images were examined to identify the presence or absence of any fracture of any part of the bony orbit (defined as any part of the orbital rim, and the lateral, superior, medial and inferior orbital walls). The presence of a fracture was reviewed only after the presence of signs and symptoms had been recorded from the clinical records to reduce bias. All records were reviewed between September 2016 and September 2017.

**Table 2:** Signs and symptoms reported by the patient or identified on examination at the time of first presentation following facial trauma

Clinical sign or symptom	
Subconjunctival haemorrhage	
Periorbital bruising	
Diplopia	
Limitation of eye movement	
Reduced sensation in the maxillary division of trigeminal nerve (V_2_)	
Change in position of globe (hypoglobus, enothalmos or proptosis)	
Decreased visual acuity	

Sensitivity, specificity, positive predictive value (PPV) and negative predictive value (NPV) were calculated for each sign and symptom for prediction of the presence of a fracture on CT using Excel (Microsoft; NM, USA). 95% confidence intervals were calculated for each statistic.

## Results

Forty-seven patients were included in the analysis, and of these 35 (74.5%) had a fracture involving any part of the bony orbit. The mean age was 40.6 years (SD 18.1) and 41 patients (87.2%) were male. The mean number of signs and symptoms recorded in patients with a fracture was 2.5 (SD: 1.4) and for patients without a fracture this was 1.1 (SD: 1.0). Findings are summarised in Table 3. Sensitivity, specificity, PPV, and NPV for each sign and symptom are summarised in Table 4.

**Table 3:** Signs and symptoms of orbital fracture recorded in the clinical notes, for patients with and without a fracture of the orbit as diagnosed by CT

	CT positive		CT negative	
	Positive, *n*	Negative, *n*	Positive, *n*	Negative, *n*
SC haem.	16	19	1	11
Periorbital bruise	26	9	7	5
Diplopia	15	20	2	10
Limitation	9	26	2	10
V_2_ numbness	9	26	1	11
Globe position	6	29	0	12
Acuity	7	28	0	12

**Table 4:** Value of signs and symptoms of orbital fractures at predicting an orbital fracture on CT. 95%CI, 95% confidence intervals

	Sensitivity, % (95%CI)	Specificity, % (95%CI)	PPV, % (95%CI)	NPV, % (95%CI)
SC haem.	45.7 (29.2–62.2)	91.7 (76.0–100)	94.1 (82.9–100.0)	36.7 (19.4–53.9)
Periorbital bruise	74.3 (59.8–88.8)	41.7 (13.8–69.6)	78.8 (64.8–92.7)	35.7 (10.6–60.8)
Diplopia	42.9 (26.5–59.3)	83.3 (62.3–100.0)	88.2 (72.9–100.0)	33.3 (16.5–50.2)
Limitation	25.7 (11.2–40.2)	83.3 (62.3–100.0)	81.8 (59.0–100.0)	27.8 (13.2–42.4)
V_2_ numbness	25.7 (11.2–40.2)	91.7 (76.0–100.0)	90.0 (71.4–100.0)	29.7 (15.0–44.5)
Globe position	17.1 (4.7–29.6)	100.0 (100.0–100.0)	100.0 (100.0–100.0)	29.3 (15.3–43.2)
Acuity	20.0 (6.8–33.3)	100.0 (100.0–100.0)	100.0 (100.0–100.0)	30.0 (15.8–44.2)

Because subconjunctival haemorrhage, reduced sensation in the distribution of V_2_, change in globe position and reduced visual acuity had specificity of over 90% (i.e., patients without a fracture were unlikely to have these signs), we felt it would be appropriate to rely on these in isolation to recommend including the orbits in CT in this population. As periorbital bruising, diplopia, and limitation of eye movement had a specificity of less than 90%, we felt these signs should be used in combination with other signs and symptoms and not in isolation to recommend CT. As a result, we assessed the validity of the following decision tool for recommending extending the field to include the orbits in the study population, the validity of which is summarised in Table 5:

- Any one of: unbounded subconjunctival haemorrhage, reduced sensation in the distribution of V_2_, change in position of the globe or reduced visual acuity.
- Any two of: periorbital bruise, diplopia, limited eye movement.

**Table 5:** Value of orbital fracture decision tool at predicting an orbital fracture on CT. 95%CI, 95% confidence intervals. The decision tool was defined as follows—any one of: unbounded subconjunctival haemorrhage; reduced sensation in the distribution of V_2_; change in position of the globe; reduced visual acuity or any two of periorbital bruising, diplopia, limited eye movement

	Sensitivity, % (95%CI)	Specificity, % (95%CI)	PPV, % (95%CI)	NPV, % (95%CI)
Orbital fracture decision tool	80.0 (66.8–93.3)	75.0 (50.5–99.5)	90.32 (79.9–100)	56.25 (31.9–80.6)

## Discussion

In the present exploratory study, our clinical decision tool had high sensitivity, specificity, and negative predictive value for the presence of a fracture of the orbit on CT. Although head injury is associated with the presence of facial fractures [2], this does not mean that all patients who have CT imaging to exclude brain injury should have CT imaging of the face—for many patients, there will be no additional benefit. In some cases, however, if the likely benefit of including the orbits in a planned trip to the CT scanner is identified at an early stage, doing so may save time and effort, and allow for more expedient diagnosis and discharge to take place. As discussed in the introduction, radiation dose from a scan including the head and orbits may actually be lower than if separate studies are performed. Although the American College of Radiology stipulate that the lens should not be included in CT of the brain [15], the orbits may still receive some radiation dose during head CT due to scatter, even when image acquisition extends only as far inferiorly as the superior orbital rim [16]. If the orbits then go on to be imaged in a separate examination, they will be irradiated twice compared with only once during a combined scan. Understanding which signs and symptoms are likely to be predictive of a fracture to the orbit, and thereby which would be most useful to guide clinicians to think about including the orbits when head CT is planned was the motivation for the present study.

Previous investigators have reported a greater incidence of certain clinical findings in patients with facial fractures. Intuitively, Holmgreen et al. found that the presence of facial lacerations, subconjunctival haemorrhage and periorbital bruising was significantly more likely in patients with facial fractures [17], and Barry et al. found the presence of various ophthalmological signs in 60% of patients with a fracture of the orbital floor or medial wall [18]. Looking more specifically at the value of clinical findings for predicting fractures, Timashpolsky et al. found that dental malocclusion, subconjunctival haemorrhage and cheek flatness had greatest sensitivity and specificity for mandibular, orbital floor and zygomatic complex fractures respectively [19]. These findings also had high value for predicting the need for surgery.

One assessment criteria for predicting the presence of any facial fracture that has been previously validated is the “Wisconsin Criteria.” In the initial study using this criteria, when any one of: palpable bony step; periorbital swelling or bruising; Glasgow coma scale < 14; dental malocclusion; or missing teeth were present, the authors reported sensitivity of 98.2% and NPV of 87.8% in predicting any facial fracture on CT [20]. A second retrospective study by the same authors showed similar value with sensitivity of 97.4% and NPV of 81.3% [21]. The criteria were externally validated in a third retrospective study, which found somewhat lower sensitivity of 81% and NPV of 60% [22]. On the face of things, this criteria would appear to be useful, and in the setting in which it was developed where patients usually undergo CT of the face by default, it is a useful tool to rule-out patients who are unlikely to have a fracture and thereby save unnecessary irradiation. In our setting, however, patients usually do not receive CT imaging of the face by default unless it is deemed necessary (ignoring the use of full-body CT as discussed earlier). The problem is that in the first two studies by Sitzman et al., the criteria had specificity of 22.3% [20] and 20.6% [20], and when externally validated this was 41% [22]. This means that a large number of patients without fractures would be irradiated unnecessarily if these criteria were used to recommend imaging the face in a setting where CT of the face is not performed by default. Additionally, it is likely that many facial fractures, particularly those of the mandible, can be adequately diagnosed and managed by plain film techniques alone avoiding the need for CT.

Exadaktylos et al. found that the presence of any one of subcutaneous emphysema, bony step, V_2_ numbness, diplopia, reduced acuity, or change in position of the globe had 100% specificity, but low sensitivity at 55.1% [13]. Yadav et al. derived a clinical risk score for orbital fracture which give equal weighting to the presence of tenderness, emphysema, subconjunctival haemorrhage, limited eye movement, painful eye movement, or epistaxis [23]. This score had good specificity, but low sensitivity.

The relevance of a test for the need for CT in facial trauma depends very much on the context. In the majority of previous work, patients were likely to receive CT which includes the facial skeleton by default [13, 19–22], and the value of any test is to reduce radiation exposure to patients unlikely to have a fracture whilst minimising the number of injuries that are missed by not scanning. In this context, a test with high sensitivity would mean most fractures are identified, and high NPV would mean where the test is negative patients are very unlikely to have a fracture; low specificity might be acceptable (as with the “Wisconsin criteria”) because patients would be scanned by default and it would not matter if patients without a fracture are irradiated, as they would be anyway. In the context of the authors’ unit, whereby patients do not receive head CT that includes the facial bones by default, a test with low specificity would mean a large number of patients without a fracture would be irradiated whom would otherwise have not. High PPV is important in this context, as this would mean patients with a positive test are very likely to have a fracture, and would therefore benefit from imaging they would otherwise not receive.

Our decision tool had high sensitivity at 80%, and our specificity of 75.5% was higher than that of the “Wisconsin criteria” meaning relatively fewer patients without a fracture would be unnecessarily irradiated; furthermore, we found PPV of 75.0%. In contrast to other investigators [17–22, 24, 25], we looked only at orbital fractures which as we felt that these were those most likely to benefit from CT imaging. We feel that the greatest potential value of a decision tool for orbital imaging is among clinicians who are non-specialists in the management of facial trauma, to allow them to quickly consider the likelihood of an orbital fracture prior to requesting CT of the head. This would allow them to consider extending the field to include the orbits, and thereby potentially save time and use of resources, prevent delays to diagnosis and discharge, and reduce radiation dose to the patient.

The aim of our preliminary study was to identify signs and symptoms of orbital trauma likely to have high predictive value for orbital fracture on CT. Our intention is that this can be used to design further, prospective studies to devise and validate a robust decision instrument which can be used clinically. We acknowledge that our study has a number of limitations. Firstly, the limited sample size resulted in our relatively wide reported confidence intervals, and although this means that the instrument we tested is not sufficiently well validated to be used clinically, our results demonstrate that such a tool has potential clinical utility if validated in further prospective studies. The retrospective nature of the work means that there is a risk of recording bias where clinical records are not completely accurate, or do not include all relevant information; we tried to control for this by excluding obviously incomplete records. There is also a risk of selection bias, and because of this the study population may differ from the true population of patients presenting to emergency departments, which affects the generalisability of our results. Our study population only included patients who received a CT of the facial bones, as recommended by the examining clinician; patients in this population are more likely to have facial fractures than the population of patients presenting to EDs, and this is reflected by the high fracture prevalence reported in our results. The true predictive value of our decision instrument in clinical use may therefore be lower than we have reported. For this reason, it is important that our findings are further validated in a population with a lower fracture prevalence such as those presenting to a typical ED. The study was not performed in a major trauma centre, and therefore included few polytrauma patients; however, we feel that this is probably the most appropriate intended population for any decision instrument, as whole body CT is more common in the setting of major trauma and the facial skeleton is more likely to be adequately imaged. To address these limitations, future work should seek to prospectively validate our criteria with a larger sample size in multiple settings with lower fracture prevalence, allowing greater precision and generalisability.

## Conclusion

Our results demonstrate that clinical findings suggestive of an orbital fracture have potential utility for predicting the presence of a fracture on CT; we found that change in the position of the globe, reduced visual acuity, subconjunctival haemorrhage and change in sensation in the maxillary division of the trigeminal nerve had greatest predictive value in this population. It is likely that a decision tool which incorporates multiple signs and symptoms would have greater value than relying on any one sign in isolation, and such a tool may be useful in the ED to recommend including the orbits in head CT for patients likely to benefit. This study was exploratory in nature, and further prospective studies are required in a population more applicable to a typical ED before a robust instrument can be devised for this purpose.
